# Improved performance of the artus *Mycobacterium tuberculosis* RG PCR kit in a low incidence setting: a retrospective monocentric study

**DOI:** 10.1038/s41598-017-14367-z

**Published:** 2017-10-26

**Authors:** Britta Kohlmorgen, Johannes Elias, Christoph Schoen

**Affiliations:** 1 Institute for Hygiene and Microbiology, University of Wuerzburg, Wuerzburg, Germany; 2 Present Address: Institute of Microbiology, DRK Kliniken Berlin, Berlin, Germany

## Abstract

Tuberculosis (TB) and the spread of *Mycobacterium tuberculosis* complex (MTBC) strains resistant against rifampin (RIF) and isoniazid (INH) pose a serious threat to global health. However, rapid and reliable MTBC detection along with RIF/INH susceptibility testing are challenging in low prevalence countries due to the higher rate of false positives. Here, we provide the first performance data for the artus MTBC PCR assay in a low prevalence setting. We analyze 1323 respiratory and 311 non-respiratory samples with the artus MTBC PCR assay as well as by mycobacterial culture and microscopy. We propose retesting of specimens in duplicate and consideration of a determined cycle-threshold value cut-off greater than 34, as this significantly increases accuracy, specificity, and negative predictive value without affecting sensitivity. Furthermore, we tested fourteen MTBC positive samples with the GenoType MTBDR*plus* test and demonstrate that using an identical DNA extraction protocol for both assays does not impair downstream genotypic testing for RIF and INH susceptibility. In conclusion, our procedure optimizes the use of the artus MTB assay with workload efficient methods in a low incidence setting. Combining the modified artus MTB with the GenoType MTBDR*plus* assays allows rapid and accurate detection of MTBC and RIF/INH resistance.

## Introduction

Tuberculosis (TB) still remains a major threat to global health, and providing rapid and reliable TB diagnostics is essential for immediate patient care and to meet public health needs. The detection of acid-fast bacilli (AFB) in sputum smear microscopy and, in particular, the cultivation of *Mycobacterium tuberculosis* (MTB) complex bacteria from clinical specimens are still the gold standard for the laboratory detection of TB^1^. However, microscopy lacks sensitivity and specificity, and while cultural detection of MTB complex has both a high sensitivity and specificity it is rather time consuming and requires specialized staff and laboratory equipment. Therefore, nucleic acid amplification tests (NAATs) have come into wider use for fast and direct detection of MTB complex in clinical samples, and a large number of NAATs with a high sensitivity and specificity are now commercially available^2–9^.

Particularly for low- and middle-income countries, which often suffer from a middle to high burden of disease, the World Health Organization endorsed since 2011 the use of the automated and fully integrated GeneXpert MTB/RIF (GX) assay (Cepheid, Sunnyvale, California, USA)^10–13^. As a closed system, it combines in a cartridge system DNA extraction from the clinical sample with molecular detection of MTB complex and resistance testing against the first line anti-TB drug rifampin (RIF), and it is thus particularly suitable for point-of-care use when laboratory resources are limited otherwise. Despite its ease of use and robustness, a principal drawback of such closed systems is that once the DNA is extracted, it cannot be used for other diagnostic assays like genotypic testing for other antibiotic resistances. In particular, the GX assay completely ignores isoniazid (INH) resistance, which is found in 5% to 15% of RIF-susceptible cases worldwide and has a significant impact on treatment outcome^14,15^. Consequently, it is also not suited for the timely detection of multidrug-resistant (MDR) MTB strains^16^, defined as resistance toward RIF and INH. This would be required for the rapid interruption of transmission by adequate isolation and treatment.

The artus *Mycobacterium tuberculosis* RG PCR (artus MTB) kit (QIAGEN, Hilden, Germany) is a real-time PCR assay for the detection of all members of the tuberculosis complex using the Rotor-Gene Q (QIAGEN, Hilden, Germany) system. As an open system DNA extraction is separated from MTB detection. Consequently, this allows for the combination with other NAAT-based assays such as the GenoType MTBDR*plus* strip (Hain Lifesciences, Nehren, Germany) for the genotypic resistance testing against both first-line anti-TB drugs RIF and INH directly from clinical specimens.

Unfortunately, previous performance studies including the artus MTB kit were either focussed exclusively on paraffin-embedded tissues^17–20^ or only analysed data from respiratory materials in middle to high incidence countries^21^. However, little data are available so far for low incidence countries such as Germany with a notification rate below 10 cases per 100.000 population^22,23^. The accordingly low prevalence of around 5 cases per 100.000 population (number for 2012, ref.^22^) compromises in particular the positive predictive value (PPV) of any test, even of those having a high sensitivity and specificity.

Accordingly, the aims of this study werxxe threefold: (i) to provide the first performance data for the artus MTB assay in a low TB incidence and prevalence setting using respiratory as well as non-respiratory samples, (ii) to assess approaches to increase the PPV of the artus MTB assay for routine diagnostics, and (iii) to test whether the assay can be successfully combined with the GenoType MTBDR*plus* strip (Hain Lifesciences) to allow for also rapid genotypic testing for RIF as well as INH resistance.

## Materials and Methods

### Sample collection and preparation

This retrospective monocentric study includes data from January 2011 to December 2014, gathered at the Institute for Hygiene and Microbiology, University of Wuerzburg, Germany. The institute provides diagnostic services predominantly for the University Hospital Wuerzburg, which is a tertiary hospital with 1430 hospital beds in lower Franconia, a region in northern Bavaria, Germany, with a population of around 1.3 million.

**Figure 1:**
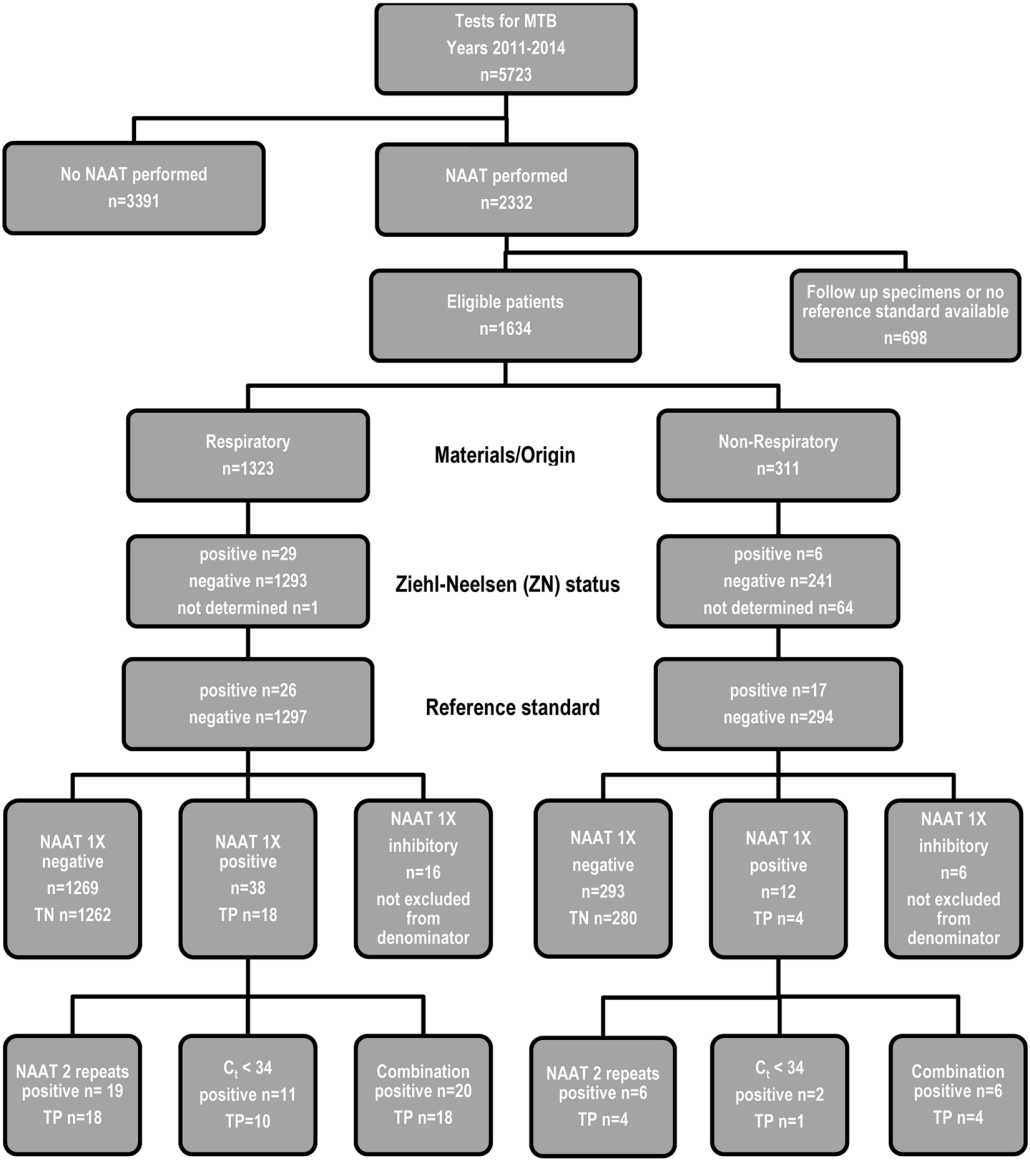
Flow diagram of patient sample types and results of the artus MTB PCR accuracy study. Abbreviations: C_t_. cycle threshold; MTB. *M. tuberculosis* bacteria. NAAT. nucleic acid amplification test (artus MTB PCR); TP. true positive; TN. true negative.

In addition to standard cultivation procedures, the artus MTB assay was only performed from respiratory and non-respiratory samples either if (a) AFB smears were positive or if (b) requested by the attendant clinician due to suspected TB. After collection, all samples were immediately transported to our laboratory and processed within 24 h. If needed, the samples were cooled at 4 °C for overnight storage, except cerebrospinal fluid (CSF), which was stored at room temperature for less than 24 hours. For the purpose of this validation study, all redundant samples from the same patients as well as all samples from patients under treatment with anti-TB drugs were excluded (Fig. 1). We note that analyses from multiple samples from the same patient of course raises the chance of finding MTBC, but the focus of this work was on a technical validation of the artus MTB assay and not on TB epidemiology.

### Ethics statement

All data analyses were performed in a patient-blinded anonymous manner. All data included in this study were taken from specimens analyzed in the diagnostic routine, and no samples were taken exclusively for the purpose of this study. Likewise, neither were the standard operating procedures affected or changed for the sake of this study, nor was the reporting of the results affected by the results of this study. The ethics committee of the Julius-Maximilians-University Würzburg (no. 20170222 02) has approved the use of clinical specimens for the validation of diagnostic tests in a patient-blinded anonymous manner without further need for obtaining informed consent from the patients.

### Microscopy, cultivation and phenotypic resistance testing

All samples except for urine, stool and genital tract specimens were examined via AFB smear using the Ziehl-Neelsen staining method (ZN) and cultivation on two solid and one liquid media (Table 1). Non-sterile material, e.g. respiratory specimens, stool, urine or gastric aspiration, were treated with N-acetyl-L-cysteine-sodium hydroxide (NALC-NaOH) before cultivation and further PCR procedures. Solid media as Stonebrink agar with polymyxin B, amphotericin B, carbapenicillin and trimethoprim (PACT) (Becton- Dickinson, Franklin Lakes, USA) and Loewenstein-Jensen-medium with glycerin and PACT (Becton-Dickinson) were used for the conventional cultivation as well as the automated liquid media system Bactec MGIT 960 (Becton-Dickinson)^1^. In line with common standards in TB diagnostics, a minimum cultivation time of eight weeks at 37 °C on solid media applied to every sample. In case of growth of AFB on solid or in fluid media PCRs and subsequent sequencing of the targets 16 S ribosomal DNA^24^ and mycobacterial gyrase B subunit DNA, *gyrB* ^25^, were performed to differentiate between the mycobacteria species of the MTB complex. Non-tuberculous mycobacteria were identified by sequencing the 16 S – 23 S rRNA intergenic transcribed spacer region according to Roth *et al*.^26^. Phenotypic antibiotic susceptibility testing was performed according to the manufacturer’s instructions with the Bactec MGIT 960 SIRE and PZA kits (Becton-Dickinson).

**Table 1:** Clinical specimens included in this study and the corresponding Ziehl-Neelsen (ZN) and culture results listed by origin.

Respiratory samples	No.	ZN positive	ZN not determined	Culture positive	Culture and ZN positive
Bronchial Secretions	845	13	0	9	5
Bronchial Lavages	258	6	0	6	4
Sputum	153	7	1	9	5
Tracheal Secretions	33	2	0	2	2
Gastric Aspirations	26	0	0	0	0
Tissues, Lung	7	1	0	0	0
Biopsy, Bronchial	1	0	0	0	0
Total	1323	29	1	26	16
**Non respiratory samples**					
Pleural Aspirations	70	0	0	4	0
Urine	68	1	63	1	1
Cerebrospinal Fluids	53	0	0	0	0
Aspirations other than Pleura	43	1	0	5	0
Tissues other than Lymphnode	34	2	0	2	0
Tissues, Lymphnode	30	2	0	2	0
Biopsies	6	0	0	2	0
Secretions	3	0	0	0	0
Ascites	2	0	0	1	0
Dialysate	1	0	0	0	0
Skin Swab	1	0	1	0	0
Total	311	6	64	17	1

### Molecular detection of MTB from clinical samples with the artus MTB assay

DNA extraction and purification was performed with 250 µl of the clinical sample, after decontamination with NALC-NaOH if appropriate, using the QIAampDNA Mini kit (QIAGEN). A Rotor-Gene Q (QIAGEN) real-time PCR cycler was used for the molecular detection of MTB complex bacteria with the artus MTB assay which is validated by the manufacturer (QIAGEN) for sputum, bronchoalveolar lavage, bronchial secretion, cerebrospinal fluid, gastric aspiration and peritoneal fluids. Results are expressed in threshold cycle (C_t_) values, expressed as an exponential increase of fluorescence compared to background fluorescence. Every C_t_ value less than or equal to the maximum cycle number of 45 was considered positive.

### Retesting of NAAT positive samples with the artus MTB

**Figure 2:**
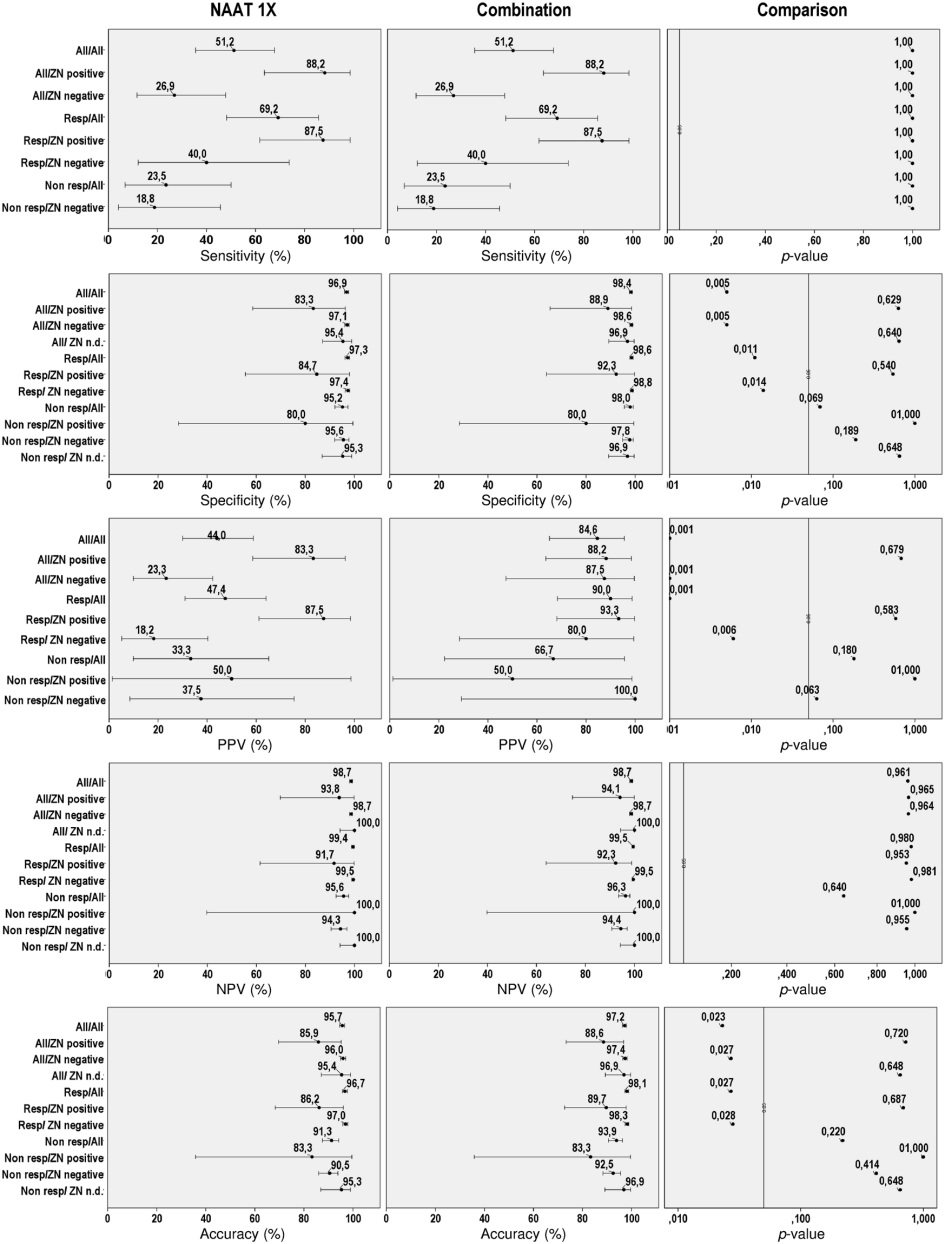
Stratified analysis of the artus MTB PCR performance data using culture as diagnostic reference. Panels on the left depict the results for the artus MTB PCR performed once according to the manufacturers’ instruction. The panels in the middle show the corresponding results for the combined application of a C_t_ cut-off in the first round and confirmation of all positive results in the second round in test-retest approach. The panels on the right display the *p*-values for the comparisons of both methods for the different parameters and sample types. The bars give the 95% confidence intervals. Abbreviations: Non resp. non-respiratory samples; n. d. no data; NPV. negative predictive value; PPV. positive predictive value. resp. respiratory samples; ZN. Ziehl-Neelsen stain.

In order to improve the PPV all artus MTB positive specimens were retested twice in parallel (“NAAT 3x” in Table 2 and Table 3) and counted as positive only if the initial positive result could be confirmed at least once. Primarily negative materials were not retested (“NAAT 1x”) and counted as “NAAT negative” (Fig. 2 and Tables 2 and 3).

**Table 2:** Stratified results for sensitivity, specificity and accuracy of the artus MTB assay using culture as diagnostic reference.

Results	Test implementation	Sensitivity				Specificity				Accuracy			
All samples		TP/P¹	%	[95%-CI]	p-value^2^	TN/N^3^	%	[95%-CI]	p-value	TN + TP/N + P	%	[95%-CI]	p-value
All (n = 1634)	NAAT1X	22/43	51.2	[35.5; 67.7]	n.a.	1542/1591	96.9	[96.0; 97.7]	n.a.	1564/1634	95.7	[94.6; 96.6]	n.a.
	NAAT3X	22/43	51.2	[35.5; 67.7]	1	1567/1591	98.5	[97.8; 99.0]	**0.003**	1598/1634	97.2	[96.2; 97.9]	**0.001**
	C_t_ cut-off = 34	11/43	25.6	[13.5; 41.2]	**0.014**	1568/1591	98.5	[97.1; 99.1]	**0.002**	1579/1634	96.6	[95.6; 97.5]	0.171
	Combination	22/43	51.2	[35.5; 67.7]	1	1566/1591	98.4	[97.7; 98.9]	**0.005**	1588/1634	97.2	[96.3; 97.9]	**0.023**
ZN Positive (n = 35)	NAAT1X	15/17	88.2	[63.6; 98.5]	n.a.	15/18	83.3	[58.6; 96.4]	n.a.	30/35	85.9	[69.7; 95.2]	n.a.
	NAAT3X	15/17	88.2	[63.6; 98.5]	1	17/18	94.4	[72.7; 99.9]	0.289	32/35	91.4	[76.9; 98.2]	0.452
	C_t_ cut-off = 34	9/17	52.9	[27.8; 77.0]	**0.023**	16/18	88.9	[65.3; 98.6]	0.629	25/35	71.4	[53.7; 85.4]	0.145
	Combination	15/17	88.2	[63.6; 98.5]	1	16/18	88.9	[65.3; 98.6]	0.629	31/35	88.6	[73.3; 96.8]	0.720
ZN Negative (n = 1534)	NAAT1X	7/26	26.9	[11.6; 47.8]	n.a.	1465/1508	97.1	[96.2; 97.9]	n.a.	1472/1534	96,0	[94.9; 97.0]	n.a.
	NAAT3X	7/26	26.9	[11.6; 47.8]	1	1487/1508	98.6	[97.9; 99.1]	**0.005**	1494/1534	97.4	[96.5; 98.1]	**0.027**
	C_t_ cut-off = 34	2/26	7.7	[0.9; 25.1]	0.067	1488/1508	98.7	[97.9; 99.2]	**0.003**	1450/1534	94.5	[93.3; 95.6]	**0.062**
	Combination	7/26	26.9	[11.6; 47.8]	1	1487/1508	98.6	[97.9; 99.1]	**0.005**	1494/1534	97.4	[96.5; 98.1]	**0.027**
ZN n.d. (n = 65)	NAAT1X	0/0	n.a.	n.a.	n.a.	62/65	95.4	[87.1; 99.0]	n.a.	62/65	95.4	[87.1; 99.0]	n.a.
	NAAT3X	0/0	n.a.	n.a.	n.a.	63/65	96.9	[89.3; 99.6]	0.640	63/65	96.9	[89.3; 99.6]	0.648
	C_t_ cut-off = 34	0/0	n.a.	n.a.	n.a.	64/65	98.5	[91.7; 99.9]	0.310	64/65	98.5	[91.7; 99.9]	0.309
	Combination	0/0	n.a.	n.a.	n.a.	63/65	96.9	[89.3; 99.6]	0.640	63/65	96.9	[89.3; 99.6]	0.648
**Respiratory samples**													
All (n = 1323)	NAAT1X	18/26	69.2	[48.2; 85.7]	n.a.	1262/1297	97.3	[96.3; 98.1]	n.a.	1280/1323	96.7	[95.7; 97.7]	n.a.
	NAAT3X	18/26	69.2	[48.2; 85.7]	1	1281/1297	98.8	[98.0; 99.3]	**0.007**	1299/1323	98.2	[97.3; 98.8]	**0.019**
	C_t_ cut-off = 34	10/26	38.4	[20.2; 59.4]	**0.020**	1281/1297	98.8	[98.0; 99.3]	**0.007**	1291/1323	97.6	[96.6; 98.3]	0.198
	Combination	18/26	69.2	[48.2; 85.7]	1	1280/1297	98.6	[97.9; 99.2]	**0.011**	1298/1323	98.1	[97.2; 98.8]	**0.027**
ZN Positive (n = 29)	NAAT1X	14/16	87.5	[61.7; 98.5]	n.a.	11/13	84.7	[55.6; 98.1]	n.a.	25/29	86.2	[68.3; 96.1]	n.a.
	NAAT3X	14/16	87.5	[61.7; 98.5]	1	13/13	100	[75.0; 100.0]	0.141	27/29	93.1	[77.2; 99.1]	0.389
	C_t_ cut-off = 34	8/16	50.0	[24.7; 75.4]	**0.020**	12/13	92.3	[63.9; 99.8]	0.540	20/29	68.9	[49.2; 84.7]	0.115
	Combination	14/16	87.5	[61.7; 98.5]	1	12/13	92.3	[63.9; 99.8]	0.540	26/29	89.7	[72.7; 97.8]	0.687
ZN Negative (n = 1293)	NAAT1X	4/10	40.0	[12.1; 73.7]	n.a.	1250/1283	97.4	[96.4; 98.2]	n.a.	1254/1293	97.0	[95.9; 97.9]	n.a.
	NAAT3X	4/10	40.0	[12.1; 73.7]	1	1267/1283	98.8	[98.0; 99.3]	**0.014**	1271/1293	98.3	[97.4; 98.9]	**0.028**
	C_t_ cut-off = 34	2/10	20.0	[2.5; 55.6]	0.330	1268/1283	98.8	[98.1; 99.3]	**0.008**	1271/1293	98.3	[97.4; 98.9]	**0.028**
	Combination	4/10	40.0	[12.1; 73.7]	1	1267/1283	98.8	[98.0; 99.3]	**0.014**	1271/1293	98.3	[97.4; 98.9]	**0.028**
ZN n.d. (n = 1)	NAAT1X	0/0	n.a.	n.a.	n.a.	1/1	100	n.a.	n.a.	1/1	100	n.a.	n.a.
	NAAT3X	0/0	n.a.	n.a.	n.a.	1/1	100	n.a.	n.a.	1/1	100	n.a.	n.a.
	C_t_ cut-off = 34	0/0	n.a.	n.a.	n.a.	1/1	100	n.a.	n.a.	1/1	100	n.a.	n.a.
	Combination	0/0	n.a.	n.a.	n.a.	1/1	100	n.a.	n.a.	1/1	100	n.a.	n.a.
**Non-respiratory samples**													
All (n = 311)	NAAT1X	4/17	23.5	[6.8; 50.0]	n.a.	280/294	95.2	[92.1; 97.4]	n.a.	284/311	91.3	[87.5; 94.2]	n.a.
	NAAT3X	4/17	23.5	[6.8; 50.0]	1	288/294	98.0	[95.6; 99.3]	0.069	292/311	93.9	[90.6; 96.3]	0.220
	C_t_ cut-off = 34	1/17	5.9	[0.2; 28.7]	0.146	287/294	97.6	[95.2; 99.0]	0.120	288/311	92.6	[89.1; 95.3]	0.555
	Combination	4/17	23.5	[6.8; 50.0]	1	288/294	98.0	[95.6; 99.3]	0.069	292/311	93.9	[90.6; 96.3]	0.220
ZN Positive (n = 6)	NAAT1X	1/1	100	n.a.	n.a.	4/5	80.0	[28.4; 99.5]	n.a.	5/6	83.3	[35.9; 99.6]	n.a.
	NAAT3X	1/1	100	n.a.	n.a.	4/5	80.0	[28.4; 99.5]	1	5/6	83.3	[35.9; 99.6]	1
	C_t_ cut-off = 34	1/1	100	n.a.	n.a.	4/5	80.0	[28.4; 99.5]	1	5/6	83.3	[35.9; 99.6]	1
	Combination	1/1	100	n.a.	n.a.	4/5	80.0	[28.4; 99.5]	1	5/6	83.3	[35.9; 99.6]	1
ZN Negative (n = 241)	NAAT1X	3/16	18.8	[4.1; 45.7]	n.a.	215/225	95.6	[92.0; 97.8]	n.a.	218/241	90.5	[86.0; 93.9]	n.a.
	NAAT3X	3/16	18.8	[4.1; 45.7]	1	220/225	97.8	[94.9; 99.3]	0.189	223/241	92.5	[88.4; 95.5]	0.414
	C_t_ cut-off = 34	0/16	0	[0.0; 20.6]	0.069	220/225	97.8	[94.9; 99.3]	0.189	220/241	91.3	[86.9; 94.5]	0.752
	Combination	3/16	18.8	[4.1; 45.7]	1	220/225	97.8	[94.9; 99.3]	0.189	223/241	92.5	[88.4; 95.5]	0.414
ZN n.d. (n = 64)	NAAT1X	0/0	n.a.	n.a.	n.a.	61/64	95.3	[86.9; 99.0]	n.a.	61/64	95.3	[86.9; 99.0]	n.a.
	NAAT3X	0/0	n.a.	n.a.	n.a.	62/64	96.9	[89.2; 99.6]	0.648	62/64	96.9	[89.2; 99.6]	0.648
	C_t_ cut-off = 34	0/0	n.a.	n.a.	n.a.	63/64	98.4	[91.6; 99.9]	0.310	63/64	98.4	[91.6; 99.9]	0.309
	Combination	0/0	n.a.	n.a.	n.a.	62/64	96.9	[89.2; 99.6]	0.648	62/64	96.9	[89.2; 99.6]	0.648

^1^TP = true positive (NAAT and reference positive); P = positive (reference positive including also inhibitory samples).

^2^p-value of a 2-sample test for equality of proportions in comparison with the standard “NAAT1x” test implementation. p-values smaller than 0.05 are printed in bold.

^3^TN = true negative (NAAT and reference negative); N = negative (reference negative).

^4^FP = false positive (NAAT positive and reference negative).

^5^FN = false negative (NAAT negative and reference positive).

**Table 3:** Stratified results for positive (PPV) and negative (NPV) predictive values of the artus MTB assay using culture as diagnostic reference.

Results	Test implementation	PPV				NPV			
All samples		TP/TP + FP^4^	%	[95%-CI]	p-value	TN/TN + FN^5^	%	[95%-CI]	p-value
All (n = 1634)	NAAT1X	22/50	44.0	[30.0; 58.8]	n.a.	1542/1562	98.7	[98.0; 99.2]	n.a.
	NAAT3X	22/25	88.0	[68.8; 97.5]	**0.001**	1567/1587	98.7	[98.1; 99.2]	0.960
	C_t_ cut-off = 34	11/13	84.6	[54.5; 98.1]	**0.008**	1568/1599	98.1	[97.3; 98.7]	0.142
	Combination	22/26	84.6	[65.1; 95.6]	**0.001**	1566/1586	98.7	[98.1; 99.2]	0.961
ZN Positive (n = 35)	NAAT1X	15/18	83.3	[58.6; 96.4]	n.a.	15/16	93.8	[69.8; 99.8]	n.a.
	NAAT3X	15/16	93.8	[69.8; 99.8]	0.347	17/18	94.4	[72.7; 99.9]	0.932
	C_t_ cut-off = 34	9/11	81.8	[48.2; 97.7]	0.917	16/23	69.6	[47.1; 86.8]	0.066
	Combination	15/17	88.2	[63.6; 98.5]	0.679	16/17	94.1	[74.3; 99.9]	0.965
ZN Negative (n = 1534)	NAAT1X	7/30	23.3	[9.9; 42.3]	n.a.	1465/1484	98.7	[98.0; 99.2]	n.a.
	NAAT3X	7/8	87.5	[47.4; 99.7]	**0.001**	1487/1506	98.7	[98.0; 99.2]	0.964
	C_t_ cut-off = 34	2/2	100	[15.8; 100.0]	**0.020**	1488/1512	98.4	[97.7; 98.9]	0.480
	Combination	7/8	87.5	[47.4; 99.7]	**0.001**	1487/1506	98.7	[98.0; 99.2]	0.964
ZN n.d. (n = 65)	NAAT1X	0/2	n.a.	n.a.	n.a.	62/62	100	[94.1; 100.0]	n.a.
	NAAT3X	0/0	n.a.	n.a.	n.a.	63/63	100	[94.3; 100.0]	1
	C_t_ cut-off = 34	0/0	n.a.	n.a.	n.a.	64/64	100	[94.4; 100.0]	1
	Combination	0/1	n.a.	n.a.	n.a.	63/63	100	[94.3; 100.0]	1
**Respiratory samples**									
All (n = 1323)	NAAT1X	18/38	47.4	[31.0; 64.1]	n.a.	1262/1269	99.4	[98.9; 99.8]	n.a.
	NAAT3X	18/19	94.7	[74.0; 99.9]	**0.001**	1281/1288	99.5	[98.9; 99.8]	0.978
	C_t_ cut-off = 34	10/11	90.9	[58.7; 99.8]	**0.010**	1281/1296	98.8	[98.1; 99.4]	0.096
	Combination	18/20	90	[68.3; 98.8]	**0.001**	1280/1287	99.5	[98.9; 99.8]	0.98
ZN Positive (n = 29)	NAAT1X	14/16	87.5	[61.2; 98.5]	n.a.	11/12	91.7	[61.5; 99.8]	n.a.
	NAAT3X	14/14	100	[76.8; 100.0]	0.171	13/14	92.9	[66.1; 98.8]	0.910
	C_t_ cut-off = 34	8/9	88.9	[51.8; 99.7]	0.918	12/19	63.2	[38.4; 83.7]	0.077
	Combination	14/15	93.3	[68.1; 99.8]	0.583	12/13	92.3	[63.9; 99.8]	0.953
ZN Negative (n = 1293)	NAAT1X	4/22	18.2	[5.2; 40.3]	n.a.	1250/1256	99.5	[99.0; 99.8]	n.a.
	NAAT3X	4/5	80.0	[28.4; 99.5]	**0.006**	1267/1273	99.5	[99.0; 99.8]	0.981
	C_t_ cut-off = 34	2/2	100	[15.9; 100.0]	**0.010**	1268/1276	99.4	[98.8; 99.7]	0.613
	Combination	4/5	80.0	[28.4; 99.5]	**0.006**	1267/1273	99.5	[99.0; 99.8]	0.981
ZN n.d. (n = 1)	NAAT1X	0/0	n.a.	n.a.	n.a.	1/1	100	n.a.	n.a.
	NAAT3X	0/0	n.a.	n.a.	n.a.	1/1	100	n.a.	n.a.
	C_t_ cut-off = 34	0/0	n.a.	n.a.	n.a.	1/1	100	n.a.	n.a.
	Combination	0/0	n.a.	n.a.	n.a.	1/1	100	n.a.	n.a.
**Non-respiratory samples**									
All (n = 311)	NAAT1X	4/12	33.3	[9.9; 65.1]	n.a.	280/293	95.6	[92.5; 97.6]	n.a.
	NAAT3X	4/6	66.7	[22.3; 95.7]	0.178	288/299	96.3	[93.5; 98.1]	0.640
	C_t_ cut-off = 34	1/2	50	[1.3; 98.7]	0.649	287/303	86.9	[82.9; 90.4]	0.632
	Combination	4/6	66.7	[22.3; 95.7]	0.180	288/299	96.3	[93.5; 98.1]	0.640
ZN Positive (n = 6)	NAAT1X	1/2	50.0	[1.3; 98.7]	n.a.	4/4	100	[39.8; 100.0]	n.a.
	NAAT3X	1/2	50.0	[1.3; 98.7]	1	4/4	100	[39.8; 100.0]	1
	C_t_ cut-off = 34	1/2	50.0	[1.3; 98.7]	1	4/4	100	[39.8; 100.0]	1
	Combination	1/2	50.0	[1.3; 98.7]	1	4/4	100	[39.8; 100.0]	1
ZN Negative (n = 241)	NAAT1X	3/8	37.5	[8.5; 75.5]	n.a.	215/229	94.3	[90.5; 96.9]	n.a.
	NAAT3X	3/3	100	[29.2; 100.0]	0.064	220/233	94.4	[90.7; 97.0]	0.955
	C_t_ cut-off = 34	0/0	n.a.	n.a.	n.a.	220/236	93.2	[89.2; 96.1]	0.632
	Combination	3/3	100	[29.2; 100.0]	0.063	220/233	94.4	[90.7; 97.0]	0.955
ZN n.d. (n = 64)	NAAT1X	0/2	n.a.	n.a.	n.a.	61/61	100	[94.1; 100.0]	n.a.
	NAAT3X	0/1	n.a.	n.a.	n.a.	62/62	100	[94.2; 100.0]	1
	C_t_ cut-off = 34	0/0	n.a.	n.a.	n.a.	63/63	100	[94.3; 100.0]	1
	Combination	0/1	n.a.	n.a.	n.a.	62/62	100	[94.2; 100.0]	1

¹TP = true positive (NAAT and reference positive); P = positive (reference positive including also inhibitory samples).

²p-value of a 2-sample test for equality of proportions in comparison with the standard “NAAT1x” test implementation. p-values smaller than 0.05 are printed in bold.

^3^TN = true negative (NAAT and reference negative); N = negative (reference negative).

^4^FP = false positive (NAAT positive and reference negative).

^5^FN = false negative (NAAT negative and reference positive).

### Genotypic resistance testing with the GenoType MTBDRplus kit

For direct detection of INH and RIF resistance we used the GenoType MTBDR*plus* line probe multiplex PCR kit (Hain Lifesciences) Version 2.0 which targets common resistance mutations in the RNA polymerase gene (*rpoB*) for RIF resistance as well as low mutations in the katalase-polymerase gene (*katG)* and the promoter region of the NADH-enoyl-ACP-reductase gene (*inhA*) leading to INH resistance. The GenoLyse kit (Hain Lifesciences) was used in parallel with the QIAampDNA Mini kit (QIAGEN) on a subset of sputum samples for DNA extraction and purification as the GenoType MTBDR*plus* PCR protocol is validated only for use with the GenoLyse kit (Hain Lifesciences). After amplification of the target sequences and reverse hybridization on test stripes containing specific probes, visualization of hybridized amplicons was carried out by an enzymatic reaction according to the manufacturer’s instructions.

### Comparison with evaluation studies using different platforms in other low incidence countries

All compared studies had to be performed with a common CE-IVD certified, commercially available platform. In addition, specimens had to be obtained in a comparable epidemiological setting: the standard used in this work is a low incidence or notification rate of <10 TB cases /100.000 population^23^. For a better comparability of study results only respiratory specimens were taken into account. A PubMed/NCBI research was performed using the search-terms: “evaluation” and “performance” with each of the chosen platforms’ names: GeneXpert MTB/RIF (Cepheid), ProbeTec ET *Mycobacterium tuberculosis complex* Direct Test (DTB) (Becton- Dickinson), COBAS Taq-Man MTB Test (Roche, Basel, Switzerland) altogether and separately in every combination. Only publications in English were included.

### Statistical analyses

Unless stated otherwise, statistical analyses were performed with the R statistical software package (version 3.2.1) and with SPSS Statistics, Version 23 (IBM, Armonk, USA), respectively. P-values for comparing NAAT results and for comparing the performance of the DNA extraction methods were calculated using a 2-sample test for equality of proportions. Analysis of deviance of a logistic regression model was used to assess the impact of the ZN staining result (“positive” or “negative”) and the sample type (“respiratory” or “non-respiratory”) on the performance (“correct” or “false” with respect to the cultural result used as reference) of the *artus M. tuberculosis* assay. Calculation of Cohens Kappa was done with the http://vassarstats.net/kappa.html homepage by Richard Lowry. Binomial confidence intervals were determined using the http://statpages.info/confint.html homepage by John C. Pezzullo. We further used the diagnostic odds ratio to define a reasonable C_t_ score cut-off value to minimize false positive results.

### Data Availability Statement

The underlying primary datasets generated during the current study are not publicly available due to (medical) confidentiality but patient-blinded excerpts are available from the corresponding author on reasonable request. Most analysed data are included in the published article.

## Results

### Sample origin and ZN status

Since the purpose of this study was a per-sample analysis of the artus MTB assay performance we only investigated primary materials from each patient. The sample mix consisted of respiratory and non-respiratory materials. While bronchial secretions and lavages were the predominant specimen type for respiratory materials, pleural aspirations, urine and CSF held the majority of non-respiratory specimens. Since the majority of NAAT requests were filed by infectious diseases units, intensive or intermediate care units or medical units, only selective materials underwent direct NAAT investigation. Consequently, sputa were the most frequent respiratory specimen in overall TB requests but not for direct NAAT investigation requested by the attendant clinician (Fig. 1)^1^.

Of the 1323 respiratory specimens analyzed by mycobacterial culture, microscopy and PCR, 26 were culture positive (2.0%) of which 16 were also ZN positive. The percentage of MTB-complex culture positive samples was significantly higher among the non-respiratory samples (n = 311, 5.5%, p < 0.01). However, there were no significant differences in the proportion of ZN positive samples between respiratory and non-respiratory specimens (29/1323 vs. 6/311, p > 0.1). Altogether, given the estimated TB point prevalence in the general German population of about ≤0.001%^22^, this suggests that clinical examination increased the pre-test TB prevalence over 1000-fold, which is largely in agreement with similar values found in other laboratories in low-prevalence countries^3^.

### Overall and stratified performance of the artus *TB* TaqMan PCR

Overall, we obtained 1564 correct, i.e. either true positive or true negative results with the artus MTB PCR using cultural detection as diagnostic reference (Fig. 2 and Table 2). Fitting the impact of the sample type (respiratory or non-respiratory) and ZN staining result (positive or negative) on the artus MTB test accuracy by a logistic regression model (p < 0.005 for both explanatory variables), analysis of deviance showed that both variables had an independent and significant impact on test accuracy (p < 0.05). The artus MTB assay showed a significantly higher accuracy for respiratory than for non-respiratory sample types and for ZN negative than for ZN positive (Fig. 2 and Table 2). The artus MTB assay showed the best performance on ZN negative, respiratory samples with 97.0% correct results, which is also the most frequently encountered sample type in routine diagnostics as outlined above.

**Figure 3:**
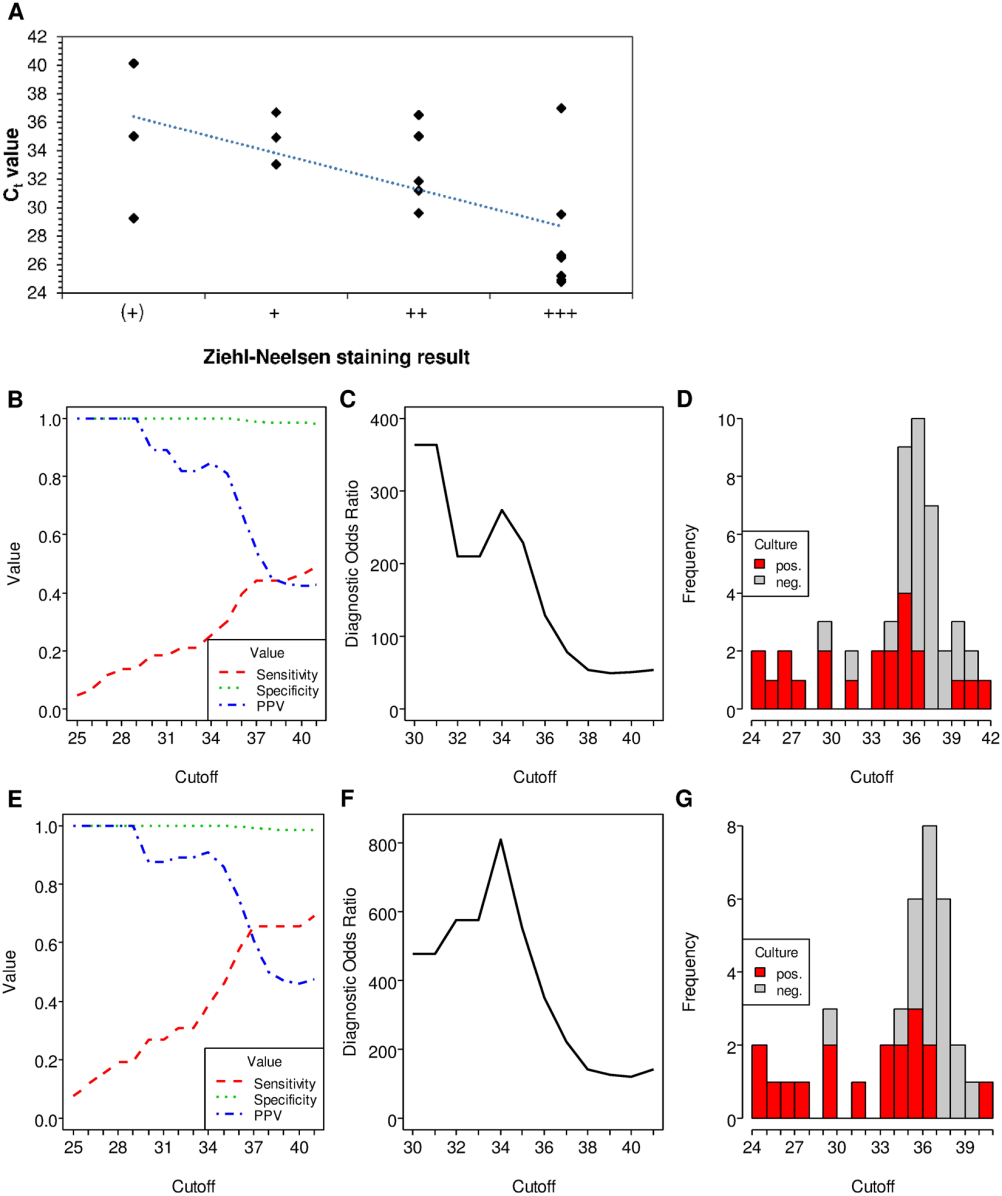
Use of threshold cycle number (C_t_) values to assess the artus MTB PCR performance. (**A**) Scatter plot depicting the threshold cycle number (C_t_) values of Ziehl-Neelsen positive samples according to the grade of acid-fast bacilli in smear microscopy. ranging from scant “(+)” to “+++”. Black triangles are individual samples and the dotted blue line gives the linear regression of the C_t_ value data. (**B**) Sensitivity, specificity and positive predictive value (PPV) (ordinate) in dependency on C_t_ cut-off values (abscissa). (**C**) Diagnostic odds ratio in dependency on the C_t_ values. (**D**) Histogram depicting the frequency of positive artus MTB test results (ordinate) in dependency on C_t_ values (abscissa). Grey: false positive NAATs (reference negative). red: true positive NAATs (reference positive). Panels (**E**) to (**F**) are identical to panels (**B**) to (**D**) except that the data are based only on the 1323 respiratory samples as validated by the manufacturer for use with the artus MTB test for calculating the diagnostic odds ratio.

Furthermore, while there was only a moderate overall concordance between the ZN staining and the artus MTB results (Cohens κ = 0.44, 95%-CI = [0.2979, 0.5735]), there was a yet significant negative correlation between the semi-quantitative number of acid-fast bacilli (ranging from “(+)” to “+++”) and the respective C_t_ value (Spearman’s rank correlation coefficient ρ = − 0.58, p < 0.05) among the ZN positive specimens (Fig. 3A).

The overall sensitivity of the artus MTB assay was rather low with 51.2% (Fig. 2 and Table 2). As expected, the sensitivity was highest in the subgroup of ZN positive specimens (88.2%) and lowest in ZN negative non-respiratory samples (18,8%). The sensitivity was significantly higher for respiratory (69.2%) than for non-respiratory samples (23.5%).

The overall specificity of the artus MTB assay was quite high with 96.9% and was highest for ZN negative and significantly lower in ZN positive respiratory samples. Overall, the specificity was only slightly higher for respiratory than for non-respiratory samples (Fig. 2 and Table 2).

In accordance with the low prevalence setting, the overall NPV was very high with 98.7% (Fig. 2 and Table 3). It was highest in the clinically most frequent subgroup of ZN negative respiratory samples (99.5%), still 95.6% for non-respiratory specimens and 98.7% irrespective of sample origin or ZN staining result.

In contrast to the excellent NPV, the overall PPV was poor with 44.0%. It was highest in the clinically highly relevant subgroup of ZN positive respiratory samples (87.5%) but was only 18.2% in the ZN negative respiratory samples. The overall PPV was non-significantly higher for respiratory than for non-respiratory sample types (47.4% vs. 33.3%) but significantly higher for ZN positive compared to ZN negative samples (83.3% vs. 23.3%), respectively.

### Retesting of NAAT positive samples with the artus MTB increases overall specificity and PPV but at the cost of a higher work load

Retesting of NAAT positive samples with the artus MTB resulted in 50 additional samples retested twice among the 1634 samples already tested once, bringing the total number of artus MTB tests to 1734.

Among the respiratory samples, this retesting approach resulted in a significant increase in the accuracy of the test to 98.2% (Fig. 2 and Table 2). A closer analysis revealed that 25 of the 50 retested samples were true positives when compared with the culture results. Twenty-two of these 25 samples considered as positives in the “NAAT 3x” approach were in turn also positive in the subsequent MTB culture, significantly increasing the overall PPV to 88% and the PPV for respiratory samples in particular to 94.7%, respectively. The increase in the PPV was most dramatic for the large group of ZN negative samples (87.5%). While there was also a concomitant significant increase in the overall specificity (98.5%), mainly stemming from the increase in the specificity in the two largest subgroups of respiratory (98.8%) and ZN negative samples (98.6%), respectively, there were no differences in the NPV and sensitivity between both approaches.

### Application of a C_t_ cut-off improves the overall specificity and PPV of the artus MTB PCR but at the expense of its sensitivity

Since there was no C_t_ cut-off defined by the manufacturer, samples with large C_t_ values were also considered as positive throughout this study.

As depicted in Fig. 3B and E, application of an increasing C_t_ cut-off resulted in an increase in the sensitivity at the expense of the specificity and PPV. The sensitivity increases with increasing C_t_ value, yet does not surpass approx. 50.0% at C_t_ = 41. The specificity slightly decreases with increasing C_t_ values, yet stayed above 98.5% at C_t_ = 41. The PPV markedly decreases with increasing C_t_ values, dropping to approx. 40.0% at C_t_ = 41. The diagnostic odds ratio showed a maximum at a C_t_ cut-off of 34, and most of the false positive test results could be found past the 34^th^ cycle (Fig. 3D and G). Applying a C_t_ cut-off of 34, a significant increase in specificity (98.5%) and PPV (84.6%) could be observed, particularly in respiratory and ZN negative but not in non-respiratory or ZN positive samples (Table 2 and Table 3). While leaving the NPV unaffected this increase came, however, at the expense of a significantly decreased sensitivity of only 25.6% at a C_t_ cut-off of 34 (p < 0.05).

### Combined application of a C_t_ cut-off in the first round and confirmation of all positive results in the second round in test-retest approach results in a significantly enhanced artus MTB PCR performance

By applying the C_t_ cut-off of 34 in the first round of artus MTB testing and confirming each positive result at least once by retesting two times in parallel (“NAAT 3x”) without such a C_t_ cut-off, the overall accuracy further increased to 97.2% when compared to the standard testing protocol as recommended by the manufacturer. As further shown in Fig. 2 and Table 2, while the sensitivity and NPV remained unaffected by this test modification, this combined approach resulted in a significant increase in the overall specificity (98.4%) and PPV (84.6%). The increase in the PPV was most dramatic for respiratory (90.0%) as well as for ZN negative (87.5%) samples.

At the same time, applying the C_t_ cut-off in the first round of testing significantly reduced the number of positive test results to be confirmed among the total of 1634 samples from 50 to 25 (p < 0.01), corresponding to only 50 additional artus MTB PCRs. In consequence, compared with the test as originally implemented by the manufacturer our test modifications resulted in a reduction of the number of false-positive patients from 28 to only 4 without altering the number of false negatives (n = 20) among the 1634 patients tested.

### Comparison of extraction methods for genotypic INH/RIF resistance testing

Since the artus MTB PCR does not allow for a simultaneous genotypic resistance testing we combined this assay with the GenoType MTBDR*plus* test which detects the most frequent mutations conferring resistance against the two first line antibiotics rifampin and isoniazid. As the latter test was validated by the manufacturer only for use with the GenoLyse DNA extraction assay, we compared the GenoType MTBDR*plus* performance after DNA extraction with the GenoLyse and the QIAampDNA mini kit in fourteen MTB complex positive clinical samples using phenotypic resistance testing as reference. We obtained identical results with both assays in eleven cases (78.6%, p < 0.001, exact binomial test), comprising four cases with correct identified susceptibilities, two cases with correct identified resistances, two cases with false negative results compared to phenotypic resistance testing and another three cases that were non-determinable. Three materials showed differing results: one case with correct susceptibilities after DNA extraction with the GenoLyse kit and a non-determinable result after DNA extraction with the QIAampDNA mini kit; two cases in which only DNA extraction with QIAampDNA mini kit resulted in the detection of correct sensitivities while after DNA extraction with the GenoLyse protocol the resistance genotype could not be determined. Together, there was no significant difference in the performance of the GenoType MTBDR*plus* assay after DNA extraction with both kits (p = 0.71, 2-sample test for equality of proportions) and the concordance between the GenoType MTBDR*plus* test results after DNA extraction with either protocol was reasonably high (Kappa = 0.65, 95-CI = [0.18,1.00]).

## Discussion

In recent years, PCR-based molecular assays have become a mainstay for the rapid detection and identification of MTB complex organisms in clinical specimens. The artus MTB is a real-time PCR-based test for which, however, performance data obtained in a low incidence/prevalence country have been lacking so far.

Using a collection of 1323 respiratory and 311 non-respiratory samples from patients with suspected TB we overall obtained 95.7% correct results using cultural detection as diagnostic reference. Whereas the overall sensitivity and PPV of the artus MTB performed once were rather poor with 51.2% and 44.0%, respectively, the specificity and NPV were quite high with 96.9% and 98.7% (Table 2, Table 3 and Fig. 2), respectively. Both, the sample type as well as ZN staining, had an independent and significant impact on the test performance. As expected, the test performed slightly better with respiratory than with non-respiratory materials and with ZN negative than with ZN positive. Accordingly, the artus MTB test performed on ZN negative, respiratory samples with 97.0% correct results and a NPV of 99.5%. For ZN positive respiratory samples, the artus MTB assay showed a comparable accuracy of 86.2% and a PPV of 87.5%. Together, the high NPV suggests that the artus MTB PCR can be used to screen respiratory samples from patients with clinically suspected TB for DNA from MTB complex bacteria in order to exclude patients tested negative from additional confirmatory testing. For ZN positive samples, the artus MTB assay has also a reasonably high PPV although positive results need further confirmatory testing.

A comparison of our results for respiratory specimens with that of other evaluation studies also performed in low incidence/prevalence settings and employing CE-IVD certified molecular assays further showed that their mean specificity and NPV were comparable to those of the artus MTB assay (96.8%, 95%-CI = [92.2%, 99.4%], and 87.0%, 95%-CI = [75.%, 98.8%], respectively)^27–30^. However, the artus MTB assay had a considerably poorer overall sensitivity and PPV when compared against this dataset (87.9%, 95%-CI = [70.2%, 97.5%], and 95.6%, 95%-CI = [87.9%, 99.9%], respectively). We note, however, that full comparability is hard to achieve due to differing study designs caused by different inclusion criteria for the samples^27,29^ and sometimes also low reproducibility among studies assessing the same test (e.g. COBASTaqManMTB test)^28,30^.

Given the lack of consistent clinical data, it remains elusive at present whether the false-positive results contributing to the low PPV were caused by residual MTB complex DNA, cross-reactivity of the artus MTB PCR with DNA from mycobacteria other than tuberculosis (MOTT) or due to other unspecific amplification reactions. In line with current recommendations^1^, we deliberately used mycobacterial culture as a reference method in order to have a consistent diagnostic “gold standard” for all 1634 patient samples and thus to avoid inconsistencies due to incomplete or missing clinical information regarding the (suspected) TB status. However, we note that this could contribute to a too high number of putatively “false” positive results of the artus MTB assay and thus the observed poor sensitivity and PPV of the molecular assay. In fact, most of the 28 artus MTB positive results that could not be confirmed by culture (Table 2 and Table 3) showed C_t_ values past the 34^th^ cycle which might be indicative of trace amounts of MTB DNA (Fig. 3D).

We note, however, that a recent validation study by Hur *et al*.^21^ performed in South Korea, having a higher TB prevalence than Germany, identified a higher C_t_ threshold of 38 cycles and could not find any link between high cycle values and false positive NAAT results.

For 11 of the 28 presumably false positive patients we had also data from interferon-gamma release assays (TSPOT.TB, Oxford Immunotec, Abingdon, Oxfordshire, UK). Of these, four were positive, indicating that they had prior exposure to MTB and therefore either a latent or active TB infection.

On the other hand, since two of these presumably false positive samples were culture positive for *M. chelonae* and *M. xenopi*, respectively, unspecific reactions are in fact likely to contribute to the number of false positives, too, and the discrepancies between the artus MTB PCR and the cultural results are likely multifactorial.

Notwithstanding these caveats, the poor PPV of the artus MTB assay compared with other validation studies^27–30^ prompted us to test whether modifications in the data analysis and/or a screening/re-testing approach would result in a significant improvement of the assay performance. In fact, by re-testing all those positive specimens in duplicate having a C_t_ value greater than 34 we could significantly increase the overall specificity, PPV and accuracy of the artus MTB assay to 98.4%, 84.6% and 97.2%, respectively, with only a moderate increase in the number of additional test runs. This reduces the number of patients that would have received an anti-MTB chemotherapy in our data set from 28 to only four, i.e. by 86%. Accordingly, per 1000 patients tested this corresponds to a decrease from 17.1 to 2.4 false positives. Given that a negative test result obtained via mycobacterial culture takes eight weeks, the approximately 30 additional artus MTB PCRs per 1000 patients tested have consequently to be weighed against over 700 daily doses of an anti-MTB combination chemotherapy along with the notification and isolation of the 13 patients that would have been erroneously tested positive over this time period.

Furthermore, the combination of the artus MTB assay for MTB detection and the GenoType MTBDR*plus* assay for genotypic detection of RIF/INH resistance seems to be a possible alternative to fully automated platforms such as the GeneXpert MTB/RIF given that sufficient laboratory resources are available^11–13^. Whereas the GeneXpert MTB/RIF is a closed system and limited to genotypic resistance testing of RIF only, the artus MTB assay is an open platform and thus allows for the combination with, e.g., the GenoType MTBDR*plus* assay for the genotypic detection of high and low level INH as well as RIF resistance patterns. Further optimization of the diagnostic workflow by utilizing the same DNA extraction method for both assays would allow a seamless integration of molecular MTB detection with genotypic RIF/INH resistance testing in respiratory as well as non-respiratory specimens. This, in turn, will facilitate optimized patient care in low incidence settings. Accordingly, further studies in low incidence countries, best in a prospective multi-center setting and including also clinical data as reference, will be required to assess the impact of such a combined testing-retesting approach on patient care and public health in a comprehensive manner.
